# Foetal Cell Transplantation for Parkinson's Disease: Focus on Graft-Induced Dyskinesia

**DOI:** 10.1155/2015/563820

**Published:** 2015-12-31

**Authors:** Elisabetta Tronci, Camino Fidalgo, Manolo Carta

**Affiliations:** Department of Biomedical Sciences, Section of Physiology, Cagliari University, SS 554, km 4.500, 09042 Monserrato, Italy

## Abstract

Transplantation of dopamine- (DA-) rich foetal ventral mesencephalic cells emerged as a promising therapy for Parkinson's disease (PD), as it allowed significant improvement of motor symptoms in several PD patients in open-label studies. However, double-blind clinical trials have been largely disappointing. The general agreement in the field is that the lack of standardization of tissue collection and preparation, together with the absence of postsurgical immunosuppression, played a key role in the failure of these studies. Moreover, a further complication that emerged in previous studies is the appearance of the so-called graft-induced dyskinesia (GID), in a subset of grafted patients, which resembles dyskinesia induced by L-DOPA but in the absence of medication. Preclinical evidence pointed to the serotonin neurons as possible players in the appearance of GID. In agreement, clinical investigations have shown that grafted tissue may contain a large number of serotonin neurons, in the order of half of the DA cells; moreover, the serotonin 5-HT1A receptor agonist buspirone has been found to produce significant dampening of GID in grafted patients. In this paper, we will review the recent preclinical and clinical studies focusing on cell transplantation for PD and on the mechanisms underlying GID.

## 1. Introduction

Parkinson's disease (PD) is the second most common neurodegenerative disorder and is characterized by intraneuronal inclusions of aggregated proteins (named Lewy bodies and Lewy neurites) and degeneration of the dopamine (DA) neurons of the substantia nigra pars compacta. The reduction of DA levels in the striatum results in the appearance of motor symptoms, including bradykinesia, rigidity, postural instability, and resting tremor. Although PD has usually been considered a pure motor disease, this pathology is now recognized as heterogeneous, and a significant variety of nonmotor symptoms such as depression, constipation, pain, and sleep disorders have been described in PD patients [1]. To date, oral administration of the DA precursor L-DOPA is the main treatment for PD, providing a significant improvement of the motor symptoms, which represent a major burden for the patients. However, with the progression of the disease, the vast majority of patients develop abnormal involuntary movements, known as L-DOPA-induced dyskinesia (LID), limiting the ability of this drug to provide a satisfactory control of the motor manifestations. Thus, alternative therapies are needed to provide good management of the symptoms also in advanced stages of disease.

Cell transplantation for treating PD was first tested as a potential therapy in 1985 [2]. In the early studies, autologous adrenal medulla cells were implanted unilaterally into the striatum of two PD patients [3]. Despite the fact that only minimal motor improvements were observed, these experiments were of great relevance as they provided proof of principle that DA levels could partially be restored by an ectopic transplantation of new DA neurons. Later on, studies in PD patients subjected to intrastriatal grafts of human foetal ventral mesencephalic (hfVM) tissue have demonstrated that transplanted DA cells can not only survive in the host striatum, but also restore striatal DA release and innervation [4–6], as shown by increased [18F]6-fluoro-l-3,4-dihydroxyphenylalanine ([18F]DOPA) uptake.

Clinical benefits in PD grafted patients have been observed in open-label trials. Accordingly, an improvement of motor symptoms, particularly of hypokinesia and on-off fluctuations, has been described in several grafted patients [4, 7–9], whereas amelioration of dyskinesia and postural instability was not as significant [8, 10, 11]. Motor improvements were accompanied in the most favourable cases by withdrawal of the L-DOPA treatment [11, 12] and paralleled by increased [18F]DOPA uptake in PET studies [5, 13, 14], suggesting that motor improvements could not be regarded as placebo effect. Interestingly, a recent study demonstrated that, in two patients with PD, the cell replacement strategy provided a long-lasting symptomatic relief, with both patients having discontinued their pharmacological treatment [15]. Accordingly, a recent study has shown that DA transporter (DAT) remains expressed in transplanted DA neurons for at least 14 years after transplantation [16], consistent with clinical findings that fetal DA neuron transplants maintain function for up to 15–18 years in patients [17]. Despite these promising results, the outcomes across the different clinical trials using hfVM tissue have been inconsistent and the procedure was abandoned in favour of deep brain stimulation.

The effect of foetal DA neuron transplants on LID has been variable; in some patients a significant reduction of LID has been observed, while in others dyskinesia has been unaffected or even made worse [4, 7, 8, 10, 18]. It was not until two double-blind placebo-controlled trials were performed [19, 20] that it was described for the first time that grafts may induce a new type of dyskinesia [21–23], known as off-state dyskinesia [19, 20] or graft-induced dyskinesia (GID), which was unrelated to the ongoing medication [19, 20, 23, 24].

GID has emerged as a potentially serious adverse effect induced by DA neuron transplants in 15 to 57% of patients (depending on the study) that have undergone cell replacement therapy [19, 20, 23]. GID phenomenology differs from LID, in being usually more repetitive, consisting in stereotypic dyskinetic movements that affected predominantly the legs [25]. GID usually appears 6 to 24 months after grafting, despite cessation of antiparkinsonian medications [20]. Although this form of dyskinesia was generally mild, in some case, it became disabling, requiring surgical intervention by deep brain stimulation [26].

## 2. Promoting Factors of GID

Development of GID has been proposed to be caused by several different mechanisms, such as extent of DA neuron degeneration [27], excessive DA release from the graft, graft size, graft placement [28–32], age [33], pretreatment with L-DOPA, severity of preoperative LID [32, 34, 35], or immunosuppression [23, 36, 37]. Moreover, the unintentional inclusion of serotonin neurons into the graft suspension has been recently considered as a risk factor for the development of GID [38, 39].

These promoting factors have been examined in the past years in GID patients. However, given the low number of grafted patients available for these studies, the development of a preclinical animal model of GID, represented by 6-OHDA-lesioned rodents subjected to striatal foetal ventral mesencephalic cell grafting, has provided a useful tool to investigate the mechanisms responsible for the appearance of GID. Studies using this animal model have shown that grafted DA neurons can survive, reinnervate the lesioned striatum, and provide significant improvement of motor function [40]. However, differently from what is observed in grafted PD patients, who show dyskinesia in the absence of any drug medication, spontaneous GID in grafted animals has been observed only sporadically. In fact, 6-OHDA-lesioned grafted rats display only mild and transient dyskinesia when exposed to nonpharmacological stimuli, such as a new environment [28, 29, 41]. Conversely, the DA-releasing agent amphetamine is able to evoke consistent rotational behavior contralateral to the lesioned grafted striatum, as well as abnormal involuntary movements, which can be evaluated with the same rating scale used for LID [36, 42–44]. Moreover, amphetamine-induced abnormal involuntary movements, similar to those described in the rat model, can also be observed in transplanted hemiparkinsonian mice [45]. Thus, amphetamine-induced dyskinesia has become the most convenient and reproducible animal model of GID [34, 46, 47].

Analysis of patient data and the use of this animal model have allowed researchers to propose several risk factors for the development of GID, although, so far, no single most important factor has been identified. No differences in striatal [18F]DOPA uptake were observed between transplanted patients who did or did not develop GID [20, 23], suggesting that GID should not be caused by excessive growth of the grafted DA neurons, or abnormalities in striatal DA reinnervation.

Conversely, in grafted rats, it has been demonstrated that the transplant size may have an impact on the induction of GID. In fact, rats with large grafts displayed more severe GID (amphetamine-induced) compared to rats with smaller grafts [29]. Moreover, graft placement may be another risk factor; indeed, in 6-OHDA-lesioned rats previously made dyskinetic by L-DOPA treatment, grafts placed in the caudal striatum, but not in the rostral striatum, significantly reduced L-DOPA-induced limb and orolingual dyskinesia but produced also more severe GID in response to amphetamine [28]. From that study it emerged that the severity of GID was correlated with a higher grafted-derived DA fiber density in the caudal striatum compared to the rostral part, suggesting that uneven grafting may generate “hot-spots” of DA release and produce an imbalance in the reinnervation of the host striatum [28, 30, 48].

Furthermore, the severity of preoperative LID was correlated with the severity of GID. Animals with severe preoperative LID receiving intrastriatal DA grafts showed pronounced reduction of LID, but at the same time all animals developed GID; by contrast, in rats with mild preoperative LID, which showed mild reduction of LID after grafting, the expression of GID was usually mild or absent [35]. This aspect is relevant as all patients who have undergone neural transplantation so far have been under treatment with L-DOPA for several years, and most of them presented LID prior to transplantation [25]. Thus, in an ongoing multicenter clinical trial funded by the European Commission (TRANSEURO), only patients who have not developed severe LID prior to grafting are being included in the study, in order to reduce the risk of development of GID.

## 3. Immune Suppression

Host immune response is one of the major debated issues in this field. Despite the fact that for many years the brain has been thought to have immune privilege, cell transplantation trials have demonstrated that this is not fully correct [49]. Accordingly, immunoinflammatory responses were observed in postmortem analysis of transplanted patients in which either no [19] or short-term low-dose immunosuppression [20, 50] was administered. Although it is still not fully demonstrated, immune protection seems to be essential for the successful engraftment of the striatal DA transplants [51], and lack of it may also be a potential risk factor for the development of GID. In fact, clinical observations have suggested that GID develops after early discontinuation of immunosuppressive therapy [20] and might be related to a reduction of graft survival caused by inflammatory processes around the graft [52]. However, another study showed that immunosuppressive therapy could be withdrawn without interfering with graft survival or motor recovery induced by transplantation [53], suggesting that the poor clinical response might be caused by a progressive DA denervation in areas outside the grafts [53]. Moreover, the neurodegeneration process may affect in a similar way both grafted and host nigral neurons, as pathological changes such as accumulation of Lewy bodies accompanied by downregulation of tyrosine hydroxylase (TH) and DAT have been observed in grafted cells several years after transplantation [54–56].

## 4. Graft Composition: The Serotonin Hypothesis

Recent studies suggest that GID development could be related to the composition of the grafted tissue [38, 39, 57]. It should be taken into consideration that DA cells are only about 5–10% of the cells within freshly dissociated VM tissue [58, 59] and that the way in which VM tissue is stored or cultured prior to transplantation might alter the graft composition in favour of non-DA cells, such as reactive astrocytes [60]. Additionally, different DA cell types are included in the transplant composition such as DA neurons of the A9 and A10 lineage. In fact, the number of DA A9 neurons in the transplant has been correlated with reduction of LID in rodents [61] due to their capacity to regulate DA release [62, 63].

The presence of serotonin neurons into the grafts and their role in the development of GID represents another important aspect that has emerged from recent preclinical and clinical observations. In fact, serotonin neurons develop in close proximity to the DA neurons of the ventral mesencephalon; since the availability of embryonic tissue has been a limiting factor in the previous trials, the landmarks of dissection have been usually broad, so to collect a high number of transplantable cells. However, this procedure caused the inclusion of a significant number of serotonin precursor cells in the graft [17].

It is well known that the serotonin neurons possess the machinery to take up exogenous L-DOPA, to convert it into DA, and store DA into vesicles. This mechanism plays a role in the release of L-DOPA-derived DA when the vast majority of the DA neurons have degenerated; indeed, it has been suggested that abnormal release of DA from the serotonin terminals may be responsible for the excessive swings in striatal synaptic DA levels, which underlie the appearance of LID [64–66]. Accordingly, higher striatal DA levels have been found in dyskinetic patients compared to nondyskinetic ones, as estimated by [11C]raclopride binding potential in PET imaging studies [67, 68]. Moreover, hyperinnervation of the serotonin system in the striatum of experimental models of LID, as well as of patients with dyskinesia, has been recently reported [69].

Thus, given the role of the serotonin neurons in the development of LID, the inclusion of this neuronal population in the grafted tissue has been proposed to be another risk factor for the development of GID [34, 42, 47]. In fact, in addition to a significant number of serotonin neurons, graft-derived striatal serotonergic hyperinnervation and unfavourable serotonin/DA transporter ratio have been observed in grafted patients [20, 38, 39].

The balance between serotonin and DA grafted neurons may be important for the severity of LID and GID. Indeed, in partial 6-OHDA-lesioned rats it has been demonstrated that serotonin-rich grafts were effective in inducing a widespread serotonergic hyperinnervation of the host striatum [27, 70, 71] and a progressive worsening of dyskinesia following L-DOPA treatment, compared to DA-rich grafts which, instead, dampened dyskinesia [42]. Conversely, LID was reduced in rats that received grafts containing a mixture of DA and serotonin neurons (in the proportion of 2 : 1, resp.), suggesting that as long as a sufficient portion of the DA innervation is maintained, serotonin innervation generated by the grafted serotonin neurons will have limited effect on the severity of LID [42]. However, the presence of serotonin cells in the graft may serve as a trigger for the induction of GID, as the serotonin neurons may take up DA released from the graft and release it away, in striatal regions with a poor DA innervation, which express supersensitive postsynaptic DA receptors [39, 46].

## 5. Pre- and Postsynaptic Mechanisms of GID

The involvement of the serotonin system in GID has been investigated in several preclinical studies. Using 6-OHDA-lesioned rats subjected to striatal VM grafting, significant dampening of GID (induced by amphetamine) was seen following treatment with very low doses of the 5-HT1A receptor agonist buspirone (doses unable to affect expression of LID in the same animal model) or combination of the 5-HT1A and 5-HT1B receptor agonists 8-OH-DPAT and CP94253, which all inhibit transmitter release from serotonergic neurons [34, 43]. These findings are in agreement with clinical studies where buspirone fully suppressed GID in grafted PD patients [68]. Interestingly, induction of serotonin neurotransmitter release by fenfluramine significantly increased GID induced by amphetamine administration in rats, while it was not able to induce GID when administered alone [43]. In a comparable context, an increase of GID was observed when the serotonin transporter (SERT) blocker fluvoxamine was added to the DAT blocker GBR12909 [29]. These results provide support for a role of the host serotonin innervation in the modulation of GID but also suggest that DA release from serotonin terminals may become detrimental only when DA release from grafted DA neurons is dysregulated.

Interestingly, Shin and colleagues [43] have demonstrated that removal of the endogenous serotonin innervation by specific toxin lesions appeared to abolish the anti-GID properties of the 5-HT1A and 5-HT1B agonists, suggesting that the effect of these drugs on GID is conceivably mediated by the activation of presynaptic host-derived receptors. Conversely, removal of the host serotonin innervation or pretreatment with a 5-HT1A receptor antagonist did not abolish the anti-GID effect of buspirone. These results led the authors to suggest that the modulatory effect of buspirone on GID may be independent of activation of either pre- or postsynaptic 5-HT1A receptors on serotonergic neurons but conceivably due to blockade of DA D2 receptors; indeed, it is known that buspirone also acts as a DA D2 receptor antagonist [72, 73]. In fact, a similar striking suppression of GID was also seen in 6-OHDA-lesioned rats after administration of the selective DA D2 receptor antagonist eticlopride or the DA D1 receptor antagonist SCH23390. Interestingly, both agonists suppressed GID in parkinsonian rats at doses unable to affect LID in dyskinetic 6-OHDA-lesioned (nongrafted) rats, suggesting that foetal VM grafts induce a striking enhancement of the antidyskinetic effect induced by DA receptor blockade [44]. It has been hypothesized that DA receptor blockade may unmask compensatory or maladaptive mechanisms that develop in the host striatum during chronic exposure to graft-derived DA. Such alterations may involve changes in the expression of DA receptors at synaptic membranes and/or modification of DA receptor signalling cascade [44]. Thus, preclinical and clinical evidence suggest that serotonergic and dopaminergic mechanisms may both play a role in the appearance of GID.

## 6. Future Strategies

Clinical studies showed the efficacy of transplantation of DA neurons in restoring motor functions in PD patients over a long-time period. However, the variability of the results and the appearance of side effects represented by GID in a significant percentage of grafted patients have hindered the pursuit of this approach.

There is now a general consensus that improved outcome and reduced risk of developing GID might derive from a refinement of the dissection method used to collect the foetal tissue for transplantation. Avoiding the inclusion of serotonin neurons, or other types of cells, might be one of the essential points. Furthermore, increasing the distribution of DA neurons throughout the striatum using multiple injection sites might optimize the coverage of the denervated striatum by DA transplanted cells and prevent “hot-spots” of DA release [5, 27]. In addition, the use of postoperative immunosuppressive treatment may also play an important role.

Better results may also derive from a more accurate selection of the patients; indeed, patients poorly responsive to L-DOPA treatment are less likely to benefit from this approach and should not be included in such studies. Moreover, performing PET imaging before surgery may be useful for the correct placement of the graft in each subject [24, 53].

Important knowledge is expected to be acquired with the ongoing double-blind clinical study using foetal cell transplantation in PD patients (TRANSEURO). This study will be important not only to provide a conclusive answer about the ability of foetal cell transplantation to ameliorate the motor symptoms of the disease, but also to pave the ground for future larger studies using stem cells.

## References

[1] Chaudhuri K. R., Healy D. G., Schapira A. H. V. (2006). Non-motor symptoms of Parkinson's disease: diagnosis and management. *Review: Literature and Arts of the Americas*.

[2] Backlund E. O., Granberg P. O., Hamberger B. (1985). Transplantation of adrenal medullary tissue to striatum in parkinsonism. First clinical trials. *Journal of Neurosurgery*.

[3] Lindvall O., Backlund E.-O., Farde L. (1987). Transplantation in Parkinson's disease: two cases of adrenal medullary grafts to the putamen. *Annals of Neurology*.

[4] Kordower J. H., Freeman T. B., Snow B. J. (1995). Neuropathological evidence of graft survival and striatal reinnervation after the transplantation of fetal mesencephalic tissue in a patient with Parkinson's disease. *The New England Journal of Medicine*.

[5] Mendez I., Sanchez-Pernaute R., Cooper O. (2005). Cell type analysis of functional fetal dopamine cell suspension transplants in the striatum and substantia nigra of patients with Parkinson's disease. *Brain*.

[6] Lindvall O. (2013). Developing dopaminergic cell therapy for Parkinson's disease-give up or move forward?. *Movement Disorders*.

[7] Widner H., Tetrud J., Rehncrona S. (1992). Bilateral fetal mesencephalic grafting in two patients with Parkinsonism induced by 1-methyl-4-phenyl-1,2,3,6-tetrahydropyridine (MPTP). *The New England Journal of Medicine*.

[8] Brundin P., Pogarell O., Hagell P. (2000). Bilateral caudate and putamen grafts of embryonic mesencephalic tissue treated with lazaroids in Parkinson's disease. *Brain*.

[9] Freed C. R., Breeze R. E., Rosenberg N. L. (1990). Transplantation of human fetal dopamine cells for Parkinson’s disease. Results at 1 year. *Archives of Neurology*.

[10] Defer G. L., Geny C., Ricolfi F. (1996). Long-term outcome of unilaterally transplanted Parkinsonian patients. I. Clinical approach. *Brain*.

[11] Wenning G. K., Odin P., Morrish P. (1997). Short- and long-term survival and function of unilateral intrastriatal dopaminergic grafts in Parkinson's disease. *Annals of Neurology*.

[12] Hagell P., Schrag A., Piccini P., Jahanshahi M., Brown R., Rehncrona S. (1999). Sequential bilateral transplantation in Parkinson's disease: effects of the second graft. *Brain*.

[13] Remy P., Samson Y., Hantraye P. (1995). Clinical correlates of [^18^F]fluorodopa uptake in five grafted parkinsonian patients. *Annals of Neurology*.

[14] Sawle G. V., Bloomfield P. M., Björklund A. (1992). Transplantation of fetal dopamine neurons in Parkinson's disease: PET [^18^F]6-L-fluorodopa studies in two patients with putaminal implants. *Annals of Neurology*.

[15] Kefalopoulou Z., Politis M., Piccini P. (2014). Long-term clinical outcome of fetal cell transplantation for Parkinson disease. Two case reports. *JAMA Neurology*.

[16] Hallett P. J., Cooper O., Sadi D., Robertson H., Mendez I., Isacson O. (2014). Long-term health of dopaminergic neuron transplants in Parkinson’s disease patients. *Cell Reports*.

[17] Mendez I., Vĩuela A., Astradsson A. (2008). Dopamine neurons implanted into people with Parkinson's disease survive without pathology for 14 years. *Nature Medicine*.

[18] Hauser R. A., Freeman T. B., Snow B. J. (1999). Long-term evaluation of bilateral fetal nigral transplantation in Parkinson disease. *Archives of Neurology*.

[19] Freed C. R., Greene P. E., Breeze R. E. (2001). Transplantation of embryonic dopamine neurons for severe Parkinson's disease. *The New England Journal of Medicine*.

[20] Olanow C. W., Goetz C. G., Kordower J. H. (2003). A double-blind controlled trial of bilateral fetal nigral transplantation in Parkinson's disease. *Annals of Neurology*.

[21] Lindvall O., Björklund A. (2004). Cell therapy in Parkinson’s disease. *NeuroRx*.

[22] Winkler C., Kirik D., Björklund A. (2005). Cell transplantation in Parkinson's disease: how can we make it work?. *Trends in Neurosciences*.

[23] Hagell P., Piccini P., Björklund A. (2002). Dyskinesias following neural transplantation in Parkinson's disease. *Nature Neuroscience*.

[24] Ma Y., Tang C., Chaly T. (2010). Dopamine cell implantation in Parkinson's disease: long-term clinical and ^18^F-FDOPA PET outcomes. *Journal of Nuclear Medicine*.

[25] Olanow C. W., Gracies J.-M., Goetz C. G. (2009). Clinical pattern and risk factors for dyskinesias following fetal nigral transplantation in parkinson's disease: a double blind video-based analysis. *Movement Disorders*.

[26] Herzog J., Pogarell O., Pinsker M. O. (2008). Deep brain stimulation in Parkinson's disease following fetal nigral transplantation. *Movement Disorders*.

[27] Carlsson T., Carta M., Muñoz A. (2009). Impact of grafted serotonin and dopamine neurons on development of L-DOPA-induced dyskinesias in parkinsonian rats is determined by the extent of dopamine neuron degeneration. *Brain*.

[28] Carlsson T., Winkler C., Lundblad M., Cenci M. A., Björklund A., Kirik D. (2006). Graft placement and uneven pattern of reinnervation in the striatum is important for development of graft-induced dyskinesia. *Neurobiology of Disease*.

[29] Lane E. L., Winkler C., Brundin P., Cenci M. A. (2006). The impact of graft size on the development of dyskinesia following intrastriatal grafting of embryonic dopamine neurons in the rat. *Neurobiology of Disease*.

[30] Ma Y., Feigin A., Dhawan V. (2002). Dyskinesia after fetal cell transplantation for parkinsonism: a PET study. *Annals of Neurology*.

[31] Maries E., Kordower J. H., Chu Y. (2006). Focal not widespread grafts induce novel dyskinetic behavior in parkinsonian rats. *Neurobiology of Disease*.

[32] Steece-Collier K., Soderstrom K. E., Collier T. J., Sortwell C. E., Maries-Lad E. (2009). Effect of levodopa priming on dopamine neuron transplant efficacy and induction of abnormal involuntary movements in Parkinsonian rats. *The Journal of Comparative Neurology*.

[33] Collier T. J., O'Malley J., Rademacher D. J. (2015). Interrogating the aged striatum: robust survival of grafted dopamine neurons in aging rats produces inferior behavioral recovery and evidence of impaired integration. *Neurobiology of Disease*.

[34] Lane E. L., Brundin P., Cenci M. A. (2009). Amphetamine-induced abnormal movements occur independently of both transplant- and host-derived serotonin innervation following neural grafting in a rat model of Parkinson's disease. *Neurobiology of Disease*.

[35] García J., Carlsson T., Döbrössy M., Nikkhah G., Winkler C. (2011). Extent of pre-operative L-DOPA-induced dyskinesia predicts the severity of graft-induced dyskinesia after fetal dopamine cell transplantation. *Experimental Neurology*.

[36] Lane E. L. (2008). Potential cellular and regenerative approaches for the treatment of Parkinson's disease. *Journal of Neuropsychiatric Disease and Treatment*.

[37] Soderstrom K. E., Meredith G., Freeman T. B. (2008). The synaptic impact of the host immune response in a parkinsonian allograft rat model: influence on graft-derived aberrant behaviors. *Neurobiology of Disease*.

[38] Politis M. (2010). Dyskinesias after neural transplantation in Parkinson's disease: what do we know and what is next?. *BMC Medicine*.

[39] Politis M., Oertel W. H., Wu K. (2011). Graft-induced dyskinesias in Parkinson's disease: high striatal serotonin/dopamine transporter ratio. *Movement Disorders*.

[40] Björklund A. (1992). Dopaminergic transplants in experimental parkinsonism: cellular mechanisms of graft-induced functional recovery. *Current Opinion in Neurobiology*.

[41] Vinuela A., Hallett P. J., Reske-Nielsen C. (2008). Implanted reuptake-deficient or wild-type dopaminergic neurons improve on L-dopa dyskinesias without OFF-dyskinesias in a rat model of Parkinson's disease. *Brain*.

[42] Carlsson T., Carta M., Winkler C., Björklund A., Kirik D. (2007). Serotonin neuron transplants exacerbate L-DOPA-induced dyskinesias in a rat model of Parkinson's disease. *The Journal of Neuroscience*.

[43] Shin E., Garcia J., Winkler C., Björklund A., Carta M. (2012). Serotonergic and dopaminergic mechanisms in graft-induced dyskinesia in a rat model of Parkinson's disease. *Neurobiology of Disease*.

[44] Shin E., Lisci C., Tronci E. (2014). The anti-dyskinetic effect of dopamine receptor blockade is enhanced in parkinsonian rats following dopamine neuron transplantation. *Neurobiology of Disease*.

[45] Smith G. A., Heuer A., Klein A., Vinh N.-N., Dunnett S. B., Lane E. L. (2012). Amphetamine-induced dyskinesia in the transplanted hemi-parkinsonian mouse. *Journal of Parkinson's Disease*.

[46] Shin E., Tronci E., Carta M. (2012). Role of serotonin neurons in L-DOPA- and graft-induced dyskinesia in a rat model of parkinson's disease. *Parkinson's Disease*.

[47] García J., Carlsson T., Döbrössy M., Nikkhah G., Winkler C. (2011). Impact of dopamine to serotonin cell ratio in transplants on behavioral recovery and L-DOPA-induced dyskinesia. *Neurobiology of Disease*.

[48] Lindvall O., Björklund A. (2011). Cell therapeutics in Parkinson's disease. *Neurotherapeutics*.

[49] Barker R. A., Widner H. (2004). Immune problems in central nervous system cell therapy. *NeuroRx*.

[50] Kordower J. H., Styren S., Clarke M., DeKosky S. T., Olanow C. W., Freeman T. B. (1997). Fetal grafting for Parkinson's disease: expression of immune markers in two patients with functional fetal nigral implants. *Cell Transplantation*.

[51] Piquet A. L., Venkiteswaran K., Marupudi N. I., Berk M., Subramanian T. (2012). The immunological challenges of cell transplantation for the treatment of Parkinson's disease. *Brain Research Bulletin*.

[52] Hudson J. L., Hoffman A., Strömberg I., Hoffer B. J., Moorhead J. W. (1994). Allogeneic grafts of fetal dopamine neurons: behavioral indices of immunological interactions. *Neuroscience Letters*.

[53] Piccini P. (2005). Factors affecting the clinical outcome after neural transplantation in Parkinson's disease. *Brain*.

[54] Li J.-Y., Englund E., Holton J. L. (2008). Lewy bodies in grafted neurons in subjects with Parkinson's disease suggest host-to-graft disease propagation. *Nature Medicine*.

[55] Kordower J. H., Chu Y., Hauser R. A., Freeman T. B., Olanow C. W. (2008). Lewy body-like pathology in long-term embryonic nigral transplants in Parkinson's disease. *Nature Medicine*.

[56] Chu Y., Kordower J. H. (2010). Lewy body pathology in fetal grafts. *Annals of the New York Academy of Sciences*.

[57] Niccolini F., Loane C., Politis M. (2014). Dyskinesias in Parkinson's disease: views from positron emission tomography studies. *European Journal of Neurology*.

[58] Sauer H., Brundin P. (1991). Effects of cool storage on survival and function of intrastriatal ventral mesencephalic grafts. *Restorative Neurology and Neuroscience*.

[59] Zhao S., Maxwell S., Jimenez-Beristain A. (2004). Generation of embryonic stem cells and transgenic mice expressing green fluorescence protein in midbrain dopaminergic neurons. *The European Journal of Neuroscience*.

[60] Hagell P., Cenci M. A. (2005). Dyskinesias and dopamine cell replacement in Parkinson's disease: a clinical perspective. *Brain Research Bulletin*.

[61] Kuan W.-L., Lin R., Tyers P., Barker R. A. (2007). The importance of A9 dopaminergic neurons in mediating the functional benefits of fetal ventral mesencephalon transplants and levodopa-induced dyskinesias. *Neurobiology of Disease*.

[62] Isacson O., Bjorklund L. M., Schumacher J. M. (2003). Toward full restoration of synaptic and terminal function of the dopaminergic system in Parkinson's disease by stem cells. *Annals of Neurology*.

[63] Lane E. L., Björklund A., Dunnett S. B., Winkler C. (2010). Neural grafting in Parkinson's disease: unraveling the mechanisms underlying graft-induced dyskinesia. *Progress in Brain Research*.

[64] Carta M., Carlsson T., Kirik D., Björklund A. (2007). Dopamine released from 5-HT terminals is the cause of L-DOPA-induced dyskinesia in parkinsonian rats. *Brain*.

[65] Carta M., Tronci E. (2014). Serotonin system implication in L-DOPA-induced dyskinesia: from animal models to clinical investigations. *Frontiers in Neurology*.

[66] Tronci E., Fidalgo C., Carta M. (2014). *Levodopa-Induced Dyskinesia in Parkinson's Disease*.

[67] de la Fuente-Fernández R., Sossi V., Huang Z. (2004). Levodopa-induced changes in synaptic dopamine levels increase with progression of Parkinson's disease: implications for dyskinesias. *Brain*.

[68] Politis M., Wu K., Loane C. (2014). Serotonergic mechanisms responsible for levodopa-induced dyskinesias in Parkinson's disease patients. *The Journal of Clinical Investigation*.

[69] Rylander D., Parent M., O'Sullivan S. S. (2010). Maladaptive plasticity of serotonin axon terminals in levodopa-induced dyskinesia. *Annals of Neurology*.

[70] Takeuchi Y., Sawada T., Blunt S., Jenner P., Marsden C. D. (1991). Serotonergic sprouting in the neostriatum after intrastriatal transplantation of fetal ventral mesencephalon. *Brain Research*.

[71] Wright A. K., Arbuthnott G. W., Dunnett S. B. (1991). Serotonin hyperinnervation after foetal nigra or raphe transplantation in the neostriatum of adult rats. *Neuroscience Letters*.

[72] Eison A. S., Temple D. L. (1986). Buspirone: review of its pharmacology and current perspectives on its mechanism of action. *The American Journal of Medicine*.

[73] Rijnders H. J., Slangen J. L. (1993). The discriminative stimulus properties of buspirone involve dopamine-2 receptor antagonist activity. *Psychopharmacology*.

